# The reproductive system of the male and oviparous female of a model organism—the pea aphid, *Acyrthosiphon pisum* (Hemiptera, Aphididae)

**DOI:** 10.7717/peerj.7573

**Published:** 2019-09-02

**Authors:** Karina Wieczorek, Mariusz Kanturski, Cezary Sempruch, Piotr Świątek

**Affiliations:** 1 Department of Zoology, University of Silesia in Katowice, Katowice, Poland; 2 Department of Biochemistry and Molecular Biology, Siedlce University of Natural Sciences and Humanities, Siedlce, Poland; 3 Department of Animal Histology and Embryology, University of Silesia in Katowice, Katowice, Poland

**Keywords:** Accessory glands, Genital tracts, Histology, Pest species, Sexuales, Ultrastructure

## Abstract

The structure of the reproductive system of the sexual generation—males and oviparous females—of the pea aphid *Acyrthosiphon pisum* (Harris) (Hemiptera, Aphididae), a serious pest of cultivated plants of Fabaceae, was investigated. For the first time we describe the morphology, histology and ultrastructure of the reproductive system in both morphs of the sexual generation of aphids within one species, using light and fluorescent microscopy, as well as transmission and scanning electron microscopy. The results revealed that males have testes composed of three follicles fused by the upper ends of the vasa efferentia, the vasa deferentia run independently, the accessory glands are asymmetric and the ejaculatory duct shortened. Oviparous females have ovaries composed of seven ovarioles each. The lateral oviducts join to a short common oviduct connected with the unpaired spermatheca and paired accessory glands. Yolky eggs with an aggregation of symbiotic bacteria at the posterior pole are produced. Histologically, the components of genital tracts are broadly similar: the epithelial cells of the walls of the vasa deferentia and accessory glands of the male and oviparous female have secretory functions which correlate with the age of the studied morphs. We also found symbiotic bacteria within the vasa deferentia epithelial cells in males and within the cells of the lateral oviducts of females. Because the pea aphid is listed among the 14 species that are of the greatest economic importance, our results will be useful for managing aphid populations, protecting plants and ensuring global food security.

## Introduction

More than 5,000 species of aphids (Hemiptera, Aphididae) have been described to date (Favret, 2019); all of them are characterised by apomictic parthenogenesis (clonal or asexual reproduction) as the primary or exclusive mode of reproduction (Simon, Rispe & Sunnucks, 2002; Simon, Stoeckel & Tagu, 2010; Le Trionnaire et al., 2008). Parthenogenesis, which is specific to viviparous females, is switched to sexual reproduction by changes in the photoperiod and temperature and is also influenced by physiological and genetic factors in almost all aphid lineages (Tagu, Sabater-Muñoz & Simon, 2005). However, members of the sexual generation (males and oviparous females, also known as amphigonic females) are the least known morphs due to their rarity. The sexual generation of most aphids, unlike other hemipterans, is only produced for a short period of time once per year. Therefore they are not a common subject of research (Wieczorek, 2015).

The structure of the male reproductive system of aphids has been studied in about 90 species from various subfamilies (Klimaszewski, Szelegiewicz & Wojciechowski, 1973; Głowacka et al., 1974a, 1974b; Szelegiewicz & Wojciechowski, 1985; Wojciechowski, 1977; Blackman, 1987; Polaszek, 1987a, 1987b; Gautam, 1994; Wieczorek & Wojciechowski, 2004, 2005; Wieczorek, 2006, 2008a, 2008b; Nowińska, Mróz & Depa, 2017). Currently, its ultrastructure is only known in nine species of aphids (Wieczorek & Świątek, 2008, 2009; Vitale et al., 2009; Vitale, Brundo & Viscuso, 2011). While the organisation, development and functioning of the gonads of the oviparous generation is relatively well known (Büning, 1985; Leather, Wellings & Walters, 1988; Pyka-Fościak & Szklarzewicz, 2008; Michalik, 2010; Kot, 2012; Michalik et al., 2013), a comprehensive examination of the entire reproductive system (morphology and ultrastructure) has only been made in one species (Vitale & Viscuso, 2015).

The pea aphid *Acyrthosiphon pisum* (Harris), which belongs to the tribe Macrosiphini, subfamily Aphidinae, is a complex of at least 15 genetically different host races that are generally monoecious and holocyclic (no host alternation and with a sexual phase during part of the life cycle) but under some conditions (temperature and geographical location), it can be facultatively anholocyclic (without a sexual phase) (Blackman & Eastop, 2018). It is a globally distributed polyphagous species that is associated with more than 20 legume genera (Leguminosae or Fabaceae), including cultivated species such as pea (*Pisum sativum*), red clover (*Trifolium pratense*) and alfalfa (*Medicago sativa*). The pea aphid is listed among the 14 species of the greatest economic importance and can transmit more than 30 plant viruses (Blackman & Eastop, 2000; Van Emden & Harrington, 2017).

Because the species can be reared easily and morphs can be induced by manipulating conditions, *A. pisum* is considered a model organism (Ogawa & Miura, 2014). Therefore, the pea aphid is a model species that is used to study a range of biological phenomena (Brisson & Stern, 2006), including polyphenism (Brisson, Davis & Stern, 2007; Caillaud & Losey, 2010; Ogawa & Miura, 2013; Vellichirammal, Madayiputhiya & Brisson, 2016), insect-bacterial symbioses, bacteriocyte development (Wilkinson, Fukatsu & Ishikawa, 2003; Moran & Dunbar, 2006; Brinza et al., 2009; Simon et al., 2011; Tsuchida et al., 2014; Simonet et al., 2018), the transfer of genes from fungi (Moran & Jarvik, 2010), interactions with host plants and speciation (Peccoud & Simon, 2010; Sempruch et al., 2013; Nouhaud et al., 2014; Eyres et al., 2016), as well as the molecular basis of the transition between asexual and sexual reproduction (Cortes et al., 2008; Gallot et al., 2012; Le Trionnaire et al., 2012; Bickel et al., 2013; Jaquiéry et al., 2014). Moreover, *A. pisum* is the first hemimetabolous insect of which the entire genome sequence has already been identified (The International Aphid Genomics Consortium, 2010), and the genome of its primary symbiont, *Buchnera aphidicola*, has also been sequenced (Shigenobu et al., 2000).

An enormous amount of literature has been published about this species, including a study on sexual morph determination (Lamb & Pointing, 1972), a comparison of parthenogenetic and sexual oogenesis, embryogenesis (Miura et al., 2003; Bickel et al., 2013) and egg development (Shingleton, Sisk & Stern, 2003) as well as the mating competitions of winged and wingless males (Sack & Stern, 2007) and their copulatory behaviour (Huang & Caillaud, 2012). However, the lack of basic information on the reproductive system of the sexual generation (males and oviparous females) of the pea aphid is a significant gap in understanding the biology of these hemipterans, especially in the context of advanced research on its genome, development and various aspects of its biology.

Thus, the aim of this paper is to describe the reproductive system of the adult winged males and wingless oviparous females of *A. pisum* using light and electron microscopy. We present its morphology, histology and ultrastructure with particular emphasis on the genital tracts. These will be documented for the first time in both morphs of the sexual generation of aphids, within one species. Therefore, the comprehensive analysis of the structures of the reproductive system in pea aphid will be an important contribution to understanding the reproductive processes of these hemipterans.

## Materials and Methods

### Sample collection and insect rearing

Laboratory colonies of *A. pisum* were started using field-collected aphids from *Pisum sativum* in southern Poland near Gliwice (50°27′141″N 18°27′085″E) in August 2017. The subsequent steps of the study were performed under controlled conditions. The aphids were reared in plastic cages on *Pisum sativum* var Tarchalska as the host plant in Climatic chamber KKS 240/240 TOP+ with a phytotron system (POL-ECO-APARATURA SP.J., Wodzisław Śląski, Poland). Isolated colonies of pea aphids were maintained in the conditions of a short day photoperiod of 8:16 D/N, temperature 15 °C (+/−1 °C) and humidity 70% (+/−10%) in which they produced a sexual generation of oviparous females and winged males. The aphids (45 adult sexuales) were collected directly from their host plants using a fine hairbrush and placed into Eppendorf tubes containing 70% ethanol (for total preparation) or 2.5% glutaraldehyde (for histological and ultrastructural analyses). The location, sampling date and host plant name were recorded on labels that were placed on the tubes. Voucher specimens were deposited in the collection of the Department of Zoology, University of Silesia, Katowice, Poland (DZUS). Photographs of the live specimens were taken with a Sony SLT a37 camera with extension rings.

### Total preparation

The reproductive system (from seven adult males and 10 oviparous females) were dissected from whole insects, treated with tris buffered saline and stored in glycerol, examined using a Nikon SMZ 25 stereoscopic microscope and photographed using a Nikon DS-Fi2 camera. The reproductive system was imaged from different focal planes via Z-series acquisition and automatically aligned and layered. The characters were examined using a Nikon Ni-U light microscope equipped with a phase contrast system. Pictures of the morphological details and their measurements were taken with NIS-Elements D 4.50.00 64-Bit of a Nikon SMZ 25 stereoscopic microscope. The drawings were made freehand on the Nikon Ni-U light microscope using a camera lucida. Measurements are given in millimetres. For each drawing, a magnified view is provided. The terminology of the male genitalia follows Wieczorek, Płachno & Świątek (2011).

### Light and transmission electron microscopy

The insects (nine adult males and 10 oviparous females) were decapitated and immediately fixed with 2.5% glutaraldehyde in a 0.1 M phosphate buffer, pH 7.4 at room temperature for 2 days. After washing in a phosphate buffer (pH 7.4), the material was post-fixed for 2 h in 1% OsO_4_ in a phosphate buffer, pH 7.4. The post-fixed material was washed in a graded series of ethanol, which was replaced with acetone and embedded in an Epoxy Embedding Medium Kit (Sigma, St. Louis, MO, USA). Semi-thin sections (0.7 μm thick) were cut on a Leica Ultracut ultramicrotome and stained with 1% methylene blue in a 1% sodium biborate solution at room temperature for 30 s. Additionally, semi-thin sections were stained using the periodic acid schiff (PAS) method to localise the polysaccharides, bromophenol blue (BPB) for the polypeptides, and Sudan black B for the lipids. All of the sections were examined under an Olympus BX60 microscope equipped with an XC50 digital camera (Olympus, Tokyo, Japan) and cellSens Standard software (Olympus, Tokyo, Japan). Ultra-thin sections (80 nm) were cut on an RMC Power XT ultramicrotome (RMC Boeckeler, Tucson, AZ, USA). The ultra-thin sections were contrasted with uranyl acetate (30 min) and lead citrate (20 min). The contrasted sections were examined using a Hitachi H500 transmission electron microscope at 75 kV.

Additionally, we used a pre-embedding contrasting method to better visualise the membranous components of cells. The full methodology was described by Płachno et al. (2017). The only modification was fixation; four male abdomens were fixed in 2.5% glutaraldehyde in a 0.05M cacodylate buffer (pH 7.0) for 2 days. The rest of the procedure followed the protocol presented by Płachno et al. (2017). Ultra-thin sections (80 nm) were cut on an RMC Power XT ultramicrotome and were examined using a Hitachi H500 transmission electron microscope at 75 kV without contrasting.

### Scanning electron microscopy

Adult sexual morphs for Scanning electron microscopy study (two males and two oviparous females) were preserved in 70% ethanol, then transferred and kept in 6% phosphotungstic acid solution in 70% ethanol for 24 h. Dehydration was performed using a series of ethanol changes (80–96%) for 20 min each and then two changes of absolute ethanol for 30 min. The specimens were dried in a series of hexamethyldisilazane (HMDS) and absolute ethanol (ratios of 1:3, 1:2; 2:3) for 30 min each, and then by two changes of pure HMDS for 30 min each. Dry samples were mounted on aluminium stubs with double-sided adhesive carbon labels and sputter-coated with gold in a Pelco SC-6 sputter coater (Ted Pella Inc., Redding, CA, USA) (approximately 25-nanometre layer of gold). Ready samples were analysed and imaged with a Hitachi SU8010 field emission scanning electron microscope with a secondary electron detector (Hitachi High-Technologies Corporation, Tokyo, Japan) at 5.0 and 10.0 kV accelerating voltages (Kanturski, Karcz & Wieczorek, 2015; Kanturski, Akbar & Favret, 2017).

### Differential interference contrast and fluorescence microscopy

Five adult oviparous females were decapitated and fixed in 4% formaldehyde (freshly prepared from paraformaldehyde) in PBS (phosphate buffered saline, NaCl, 137 mM; KCl, 2.7 mM; Na_2_HPO_4_, eight mM; KH_2_PO_4_, 1.5 mM, pH 7.4) for 30–40 min at room temperature and washed in PBS. Then, the whole reproductive systems were dissected, mounted on microscopic slides and analysed under an Olympus BX60 microscope equipped with a Nomarski differential interference contrast. Additionally, some ovarioles were stained with DAPI (one μg/ml; Sigma-Aldrich, St. Louis, MO, USA) for 30 min in darkness in order to visualise the DNA within the cell nuclei. Whole-mounted ovarioles were observed under an Olympus BX60 epifluorescence microscope equipped with the appropriate filters, and additionally under an Olympus FV1000 confocal microscope to visualise and count nurse cell nuclei in the tropharia.

## Results

### The male reproductive system—gross morphology

**Figure 1:**
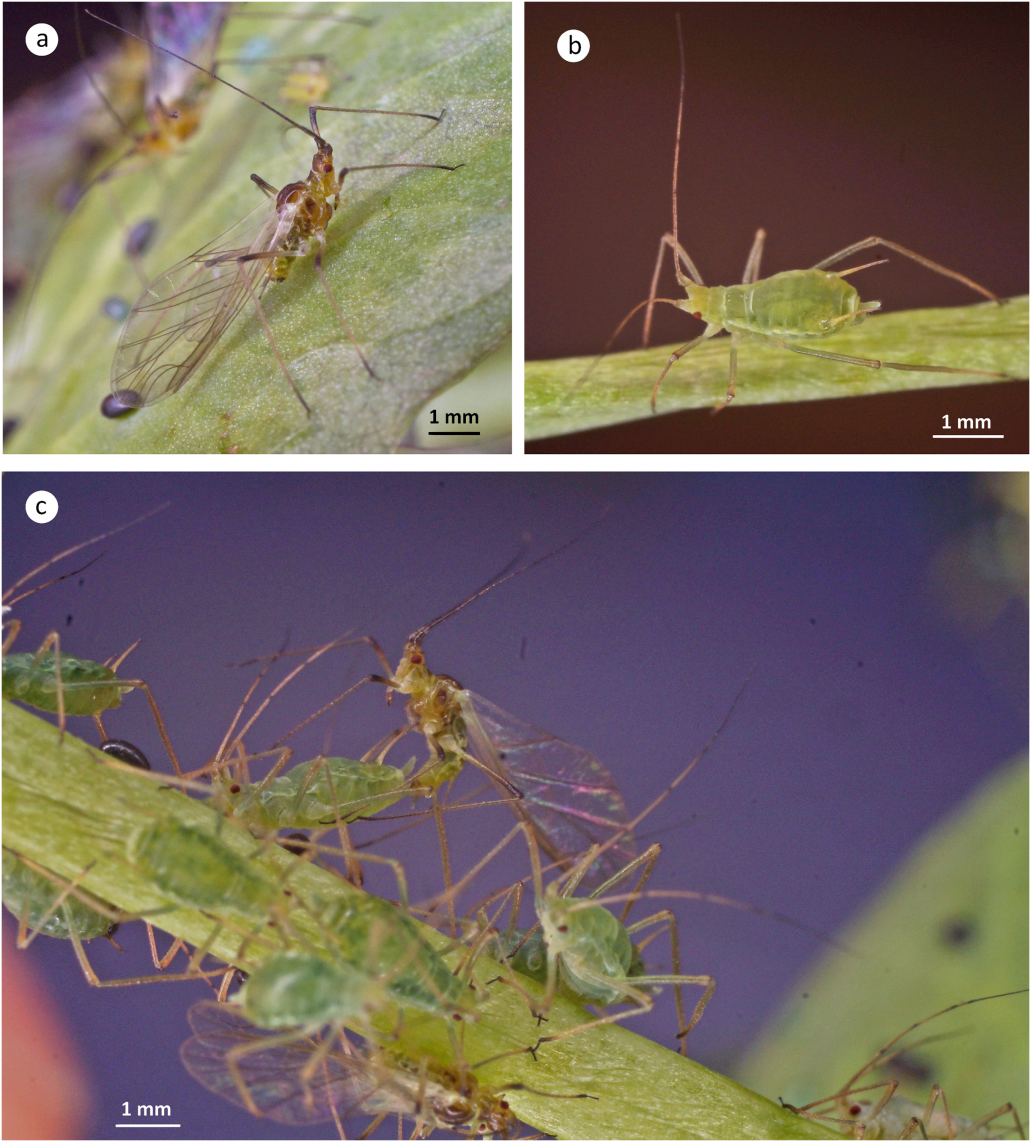
Winged male (A), wingless oviparous female (B) and copulating male and oviparous female (C) of *A. pisum*.

**Figure 2:**
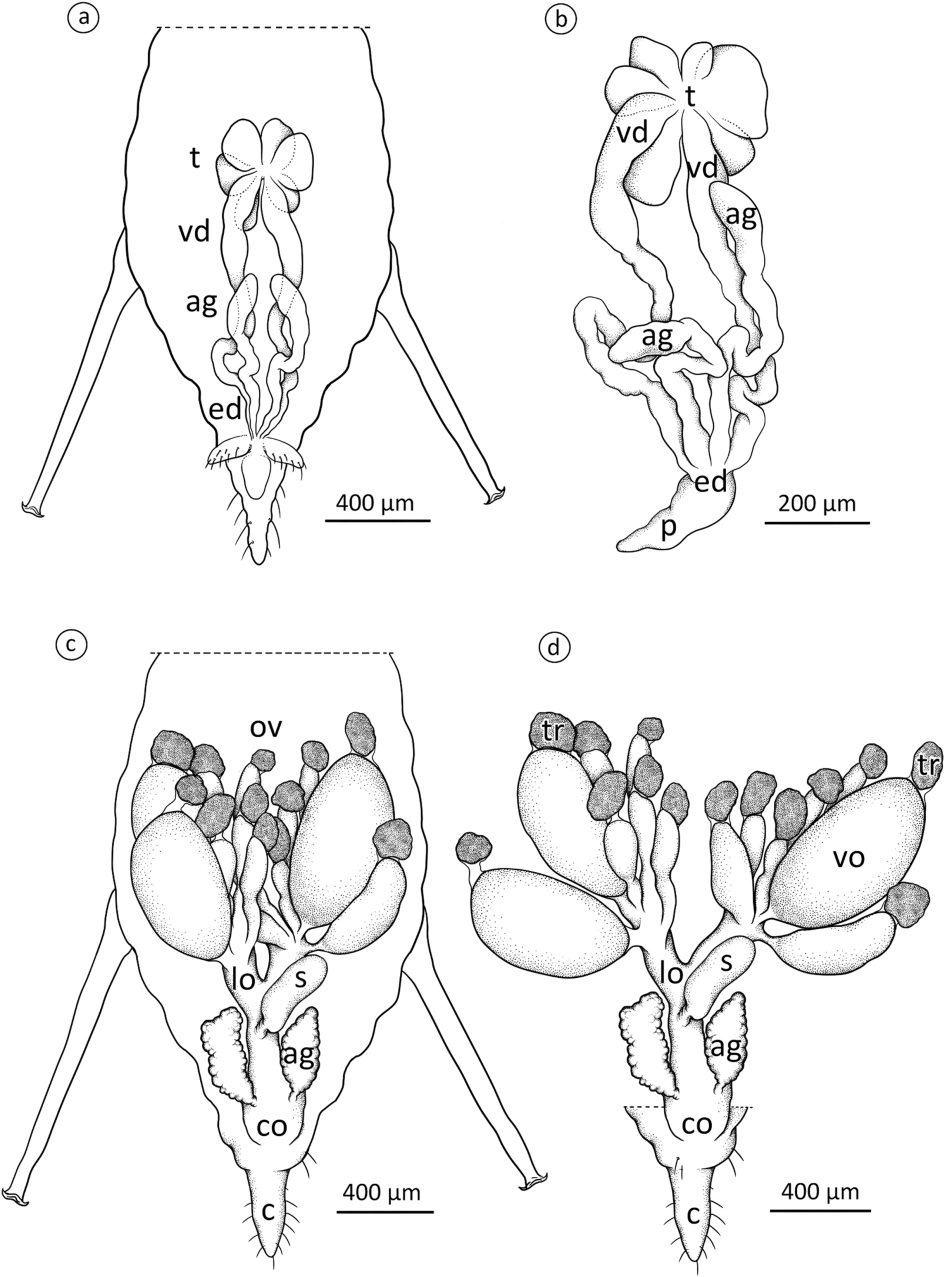
Schematic drawing presenting the localisation and morphology of male (A) and (B) and oviparous female (C) and (D) reproductive system of *A. pisum*. ag, accessory glands; c, cauda; co, common oviduct; ed, ejaculatory duct; lo, lateral oviduct; ov, ovarioles; p, penis; s, spermatheca; t, testes; tr, tropharia; vd, vasa deferentia; vo, vitellogenic oocyte. Drawing credit: Łukasz Junkiert.

**Figure 3:**
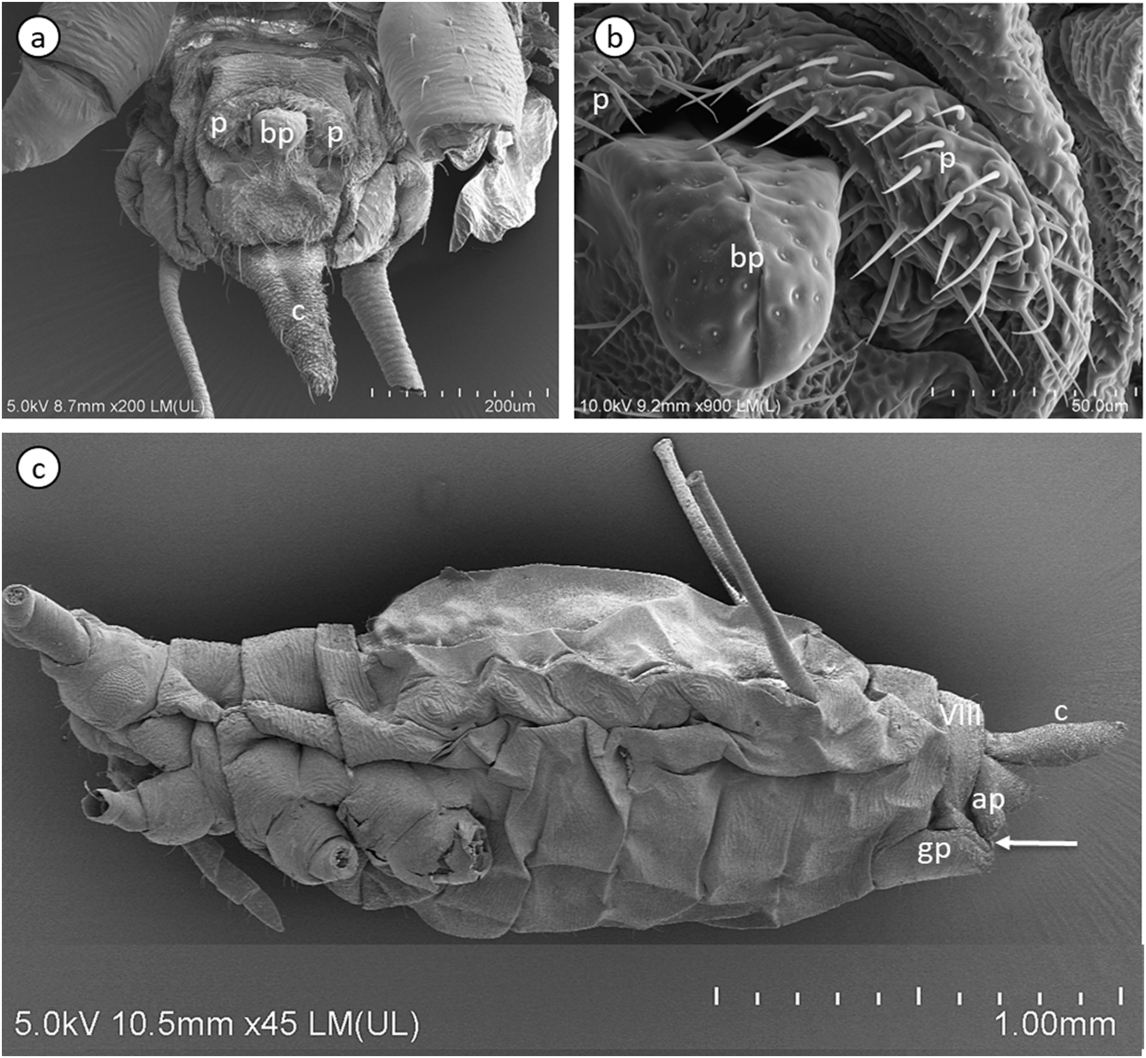
Scanning electron micrographs of the male external genitalia (A) and (B) and a whole-mounted oviparous female (C) of *A. pisum*. ap, anal plate; bp, basal parts of phallus; c, cauda; gp, genital plate; p, parameres. The arrow points to gonopore. The eighth abdominal segment (VIII) in the oviparous female is indicated.

**Figure 4:**
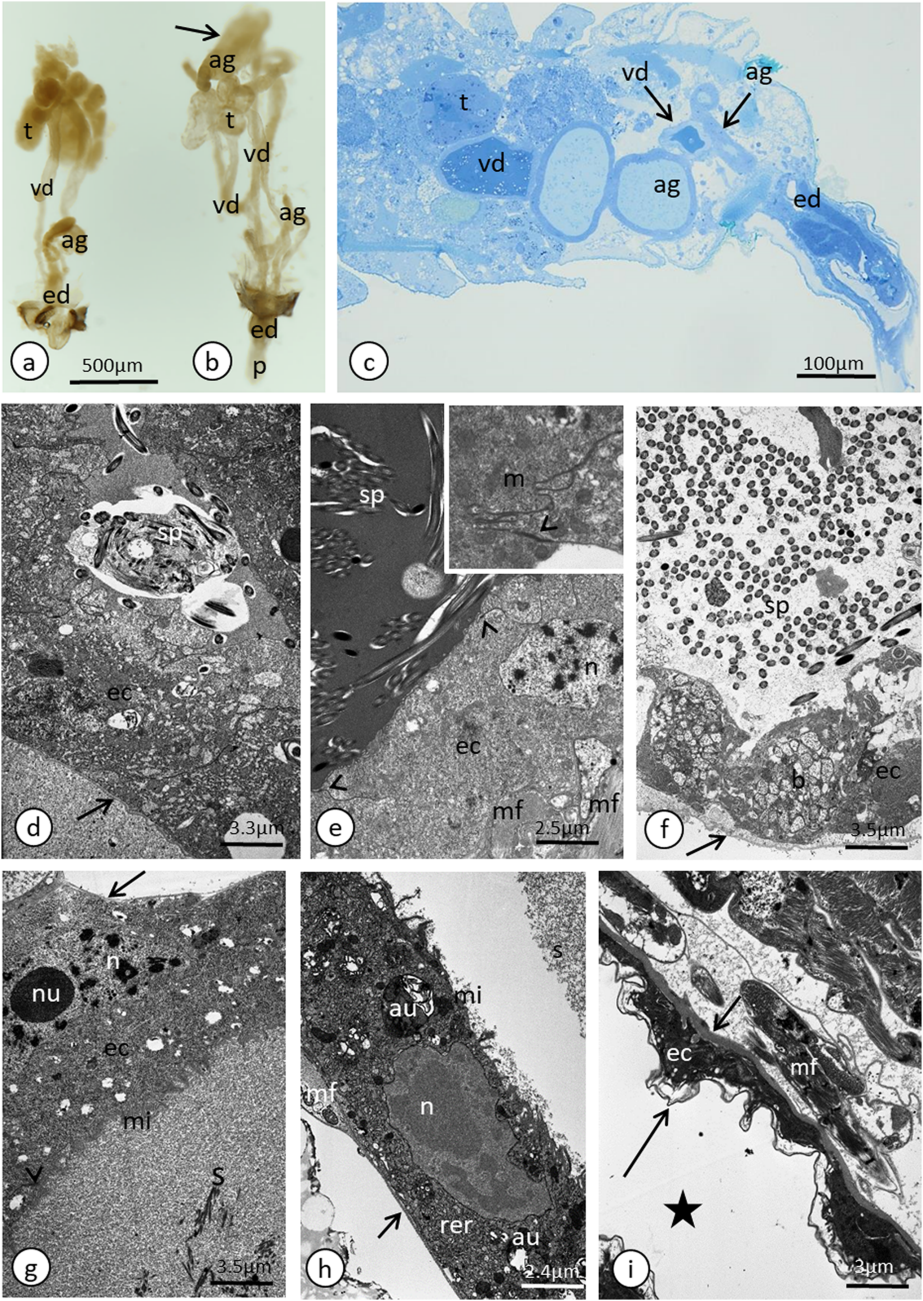
Male reproductive system of *A. pisum*. (A) Whole system in the young male. (B) Whole system in the older male. The arrow points to an expanded gland. Stereomicroscopy. (C) Abdominal segments—an epoxy semi-thin section stained with methylene blue. (D) A section through a testicular follicle. The arrow points to the basal lamina. Transmission electron microscopy (TEM). (E) and (F) Transverse sections through the vasa deferentia in its upper (E) and lower (F) portion. The arrow points to the basal lamina, the arrowheads point to the intercellular junctions of a zonula adherens type. The inset in (E) shows a cell junction at a higher magnification. TEM. (G) and (H) Transverse sections through the accessory gland. Arrows point to the basal lamina, the arrowhead points to the zonula adherens. TEM. (H) was prepared using a pre-embedding contrasting technique. (I) A transverse section through the ejaculatory duct. The short arrow points to the basal lamina, the long arrow points to the cuticle, the star points to the duct lumen. TEM. Abbreviations: ag, accessory glands; au, autophagosomes; b, bacteria; ec, epithelial cells; ed, ejaculatory duct; m, mitochondrium; mf, muscle fibres; mi, microvilli; n, nucleus; nu, nucleolus; rer, rough endoplasmic reticulum; s, secretion; sp, spermatozoa; t, testis; vd, vasa deferentia.

The internal reproductive system of the adult winged male of *A. pisum* (Fig. 1A) runs parallel to the longitudinal axis of the body in the middle part of the abdomen (Fig. 2A) and it is supplemented by the external genitalia (Figs. 3A and 3B). It is composed of paired testes, vasa efferentia, vasa deferentia, accessory glands and an ejaculatory duct (Figs. 2B, 4A, 4B and 4C). Its total length is about 1.10–1.26 mm long. Testes, which each have three follicles fused by the upper ends of the vasa efferentia, are located in the central part of the abdomen on its dorsal side in the area of abdominal segments III–IV (Fig. 2A). Oval follicles (0.12–0.19 mm long and 0.05–0.07 mm wide) are arranged in a rosette; at least two of them with apices directed towards the abdomen usually overlap. The follicles are connected to the vasa deferentia by the short vasa efferentia. The vasa deferentia run independently along the entire length and their total length is about 0.86–0.93 mm long. Their diameter is not stable—in some conditions, it can be greatly expanded to 0.05 mm in the part nearer to the testes, while in the middle and the lower part, their diameter is reduced by half (Figs. 2B, 4A and 4B). Further from the testes, they are looped in the area of abdominal segments VI–VII. The accessory glands, which are located in the central part of the abdomen on its ventral side in the area of abdominal segments VI–VIII, are club-shaped and elongated (Fig. 2A). Their total length is about 0.66–0.95 mm, but in some conditions, at least one gland is greatly elongated and its length almost equals the length of the entire reproductive system (Fig. 4B). The upper, club-shaped part is about 0.07 mm wide; the further diameter is reduced to 0.02 mm (Fig. 2B). Depending on the age of the individual and its physiological condition, the testes, diameter of the upper part of the vasa deferentia and the accessory glands change in size. In younger individuals (just after the final moulting), the testes usually are several times larger than the accessory glands and the part of the vasa deferentia nearer to the testes is not expanded. In older specimens, the testes are smaller and glands are greatly enlarged, similarly to the upper part of the vasa deferentia (Fig. 4B). The accessory glands and the vasa deferentia run separately and open into a shortened ejaculatory duct in their apical part—the vasa deferentia laterally on the dorsal side, and the accessory glands centrally on the ventral side (Figs. 2A and 2B). The external genitalia of the winged male of the pea aphid are not modified and are located on the ventral side of the abdomen (Fig. 3A). The parameres are large and lobate with numerous, rather long setae on their entire surface. The basal parts of the phallus are long and tongue-shaped. The scanning electron micrograph shows numerous circular pits that are distributed on their entire area (Fig. 3B). The phallus is housed among them and inverted during mating (Fig. 1C).

### Histological and ultrastructural properties

In the most of the studied adult male specimens that were used for the ultrastructural analyses, the process of spermatogenesis was not observed and no consecutive stages of sperm development (i.e. spermatogonia, spermatocytes and spermatids) were found within the follicles. However, in some, most likely the young males, cysts with developing spermatids were observed (Fig. S1). Separate studies that are devoted to *A. pisum* spermatogenesis and the sperm ultrastructure using the larval stages are currently being conducted and will be presented in the future.

In the studied adult specimens, the testicular follicles do not contain cysts with developing sperm; instead some chaotically oriented spermatozoa occur inside the follicle lumen (Fig. 4D). The follicle wall is formed by one layer of cuboid epithelial cells (Fig. 4D), standing on a thin basal lamina, and the cell cytoplasm is rich in endoplasmic reticulum (Fig. 4D). The apical portions of these epithelial cells are joined via complexes of cell junctions consisting of zonula adherens that lie at the apical part of the cell membranes (Fig. 4D) and septate junctions that are located more distally (Fig. S2). Some muscle strands are loosely associated with the testicular follicles; however, they do not form a clear musculature and some portions of the follicles are not associated with any muscles (Fig. 4D). The vasa deferentia wall and the wall of the accessory glands are also composed of a layer of cuboid epithelial cells with a similar organisation to the abovementioned cells that constitute the follicle wall (Figs. 4E–4H). Generally, the epithelial cells that form the vasa deferentia and accessory glands have a secretory character. Their cytoplasm is enriched in endoplasmic reticulum (Figs. 4D, 4F and 4G), which is especially clear in the sections that were prepared by the pre-embedding contrasting technique (Fig. 4H). On the other hand, no secretory vesicles are present in their cytoplasm, probably because the cells had already finished the production of secretions. Moreover, within the epithelial cells of the vasa deferentia and accessory glands, some membranous structures resembling autophagosomes were observed (Fig. 4H). In some cells of the vasa deferentia, symbiotic bacteria harbour their cytoplasm (Fig. 4F). The apical membrane of the epithelial cells may form short microvilli, while the lateral cell membranes are joined by cell junctions of zonula adherens and septate junction types (Figs. 4E and 4G). The microvilli of some of the epithelial cells of the vasa deferentia tightly encompassed single spermatozoa (Fig. S3). The lumen of the ducts is filled with a substance that has a moderate (accessory glands) or dense (vasa deferentia) character (Figs. 4C, 4E and 4G). A histochemical analysis revealed that the substance contains proteins (positive BPB staining) and is PAS positive (Fig. S4). Within the vasa deferentia lumen, some chaotically oriented bunches of spermatozoa can be observed (Fig. 4E); however, they are more abundant in the lower parts of these tracts close to the ejaculatory ducts (Fig. 4F). The musculature enveloping the vasa deferentia and accessory glands seems to be better developed than the one that is associated with testicular follicles (Figs. 4E–4G). The ejaculatory duct is formed from flattened epithelial cells standing on a relatively thick basal lamina and is covered by a cuticle (Fig. 4I). The cytoplasm of the epithelial cells is very dense and does not show any signs of synthetic activity (Fig. 4I). The ejaculatory duct is enveloped by muscles that are arranged circularly and longitudinally (Fig. 4I). Its lumen is narrow and no spermatozoa were observed within it (Fig. 4I).

### The oviparous female reproductive system—gross morphology

**Figure 5:**
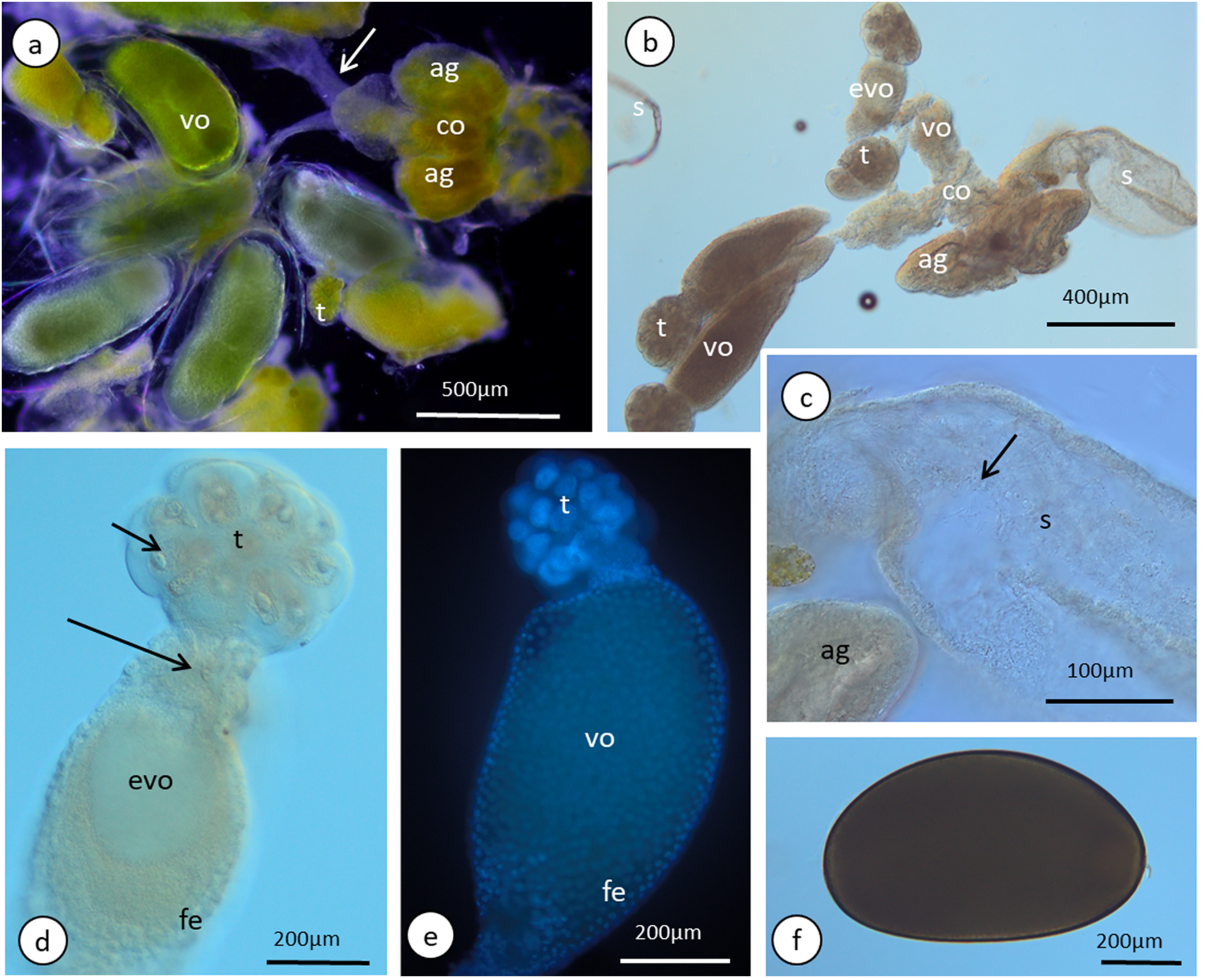
Gross morphology of the oviparous female reproductive system of *A. pisum*. (A) and (B) The reproductive system seen in a dark field (A) and under differential interference contrast (B). The arrows point to the lateral oviduct. (C) A magnified view of the lower portion of the reproductive tract. The arrow point to the spermatozoa. Light microscopy (LM), differential interference contrast. (D) and (E) Ovarioles seen under differential interference contrast (D) and in fluorescence microscopy after DAPI staining (E). The short arrow points to the nucleus of a nurse cell, the long arrow point to the nutritive cord. (F) An egg seen under differential interference contrast. Abbreviations: ag, accessory glands; co, common oviduct; evo, early vitellogenic oocyte; fe, follicular epithelium, s, spermatheca; t, tropharia; vo, vitellogenic oocytes.

The reproductive system of the adult wingless oviparous female of *A. pisum* (Fig. 1B) tightly fills the abdomen (Fig. 2C). It is composed of paired ovaries and paired lateral oviducts that join to form a short common oviduct. Additionally, the unpaired spermatheca and paired accessory glands are connected to a common oviduct (Figs. 2D, 5A and 5B). The total length of the female reproductive system is about 1.67–1.80 mm. The ovaries are meroistic telotrophic and consist of seven ovarioles. They are located in the area of abdominal segments II–V (Fig. 2C). Each ovariole is composed of a growing oocyte that is equipped with a distal tropharium (trophic chamber) that ends with an inconspicuous terminal filament. Depending on the degree of maturity, individual ovarioles vary in length from 0.33 mm long in the youngest up to a maximum of 0.8 mm long in the more advanced stages of oocyte maturation. The tropharia are usually small, spherical, 0.1–0.21 mm long and 0.16–0.21 mm wide. The paired lateral oviducts, about 0.55 mm long, join a short common oviduct that is about 0.35 mm long. The accessory glands are located in the central part of the abdomen on the ventral side in the area of abdominal segments VII–VIII (Fig. 2C). Their total length is about 0.32–0.41 mm, but in some conditions, their shape can change. In oviparous females the accessory glands are smaller, with a strongly corrugated surface prior to copulation (Fig. 2D), whereas they are lobate with a smooth surface in individuals after copulation (Fig. 5B). The spermatheca is an unpaired sac-like structure (Figs. 5A and 5B) in which the sperm is stored (Fig. 5C). Its diameter varies in individuals before (Fig. 5A) and after (Fig. 5B) copulation, and in the latter it is about 0.65 mm long. The accessory glands open into the common oviduct laterally, whereas the spermatheca opens into in its apical part. The female genital aperture is transverse and is located on the ventral side of the abdomen between the anal and genital plates and it is not equipped with any additional appendages (Fig. 3C).

### Ovarioles

**Figure 6:**
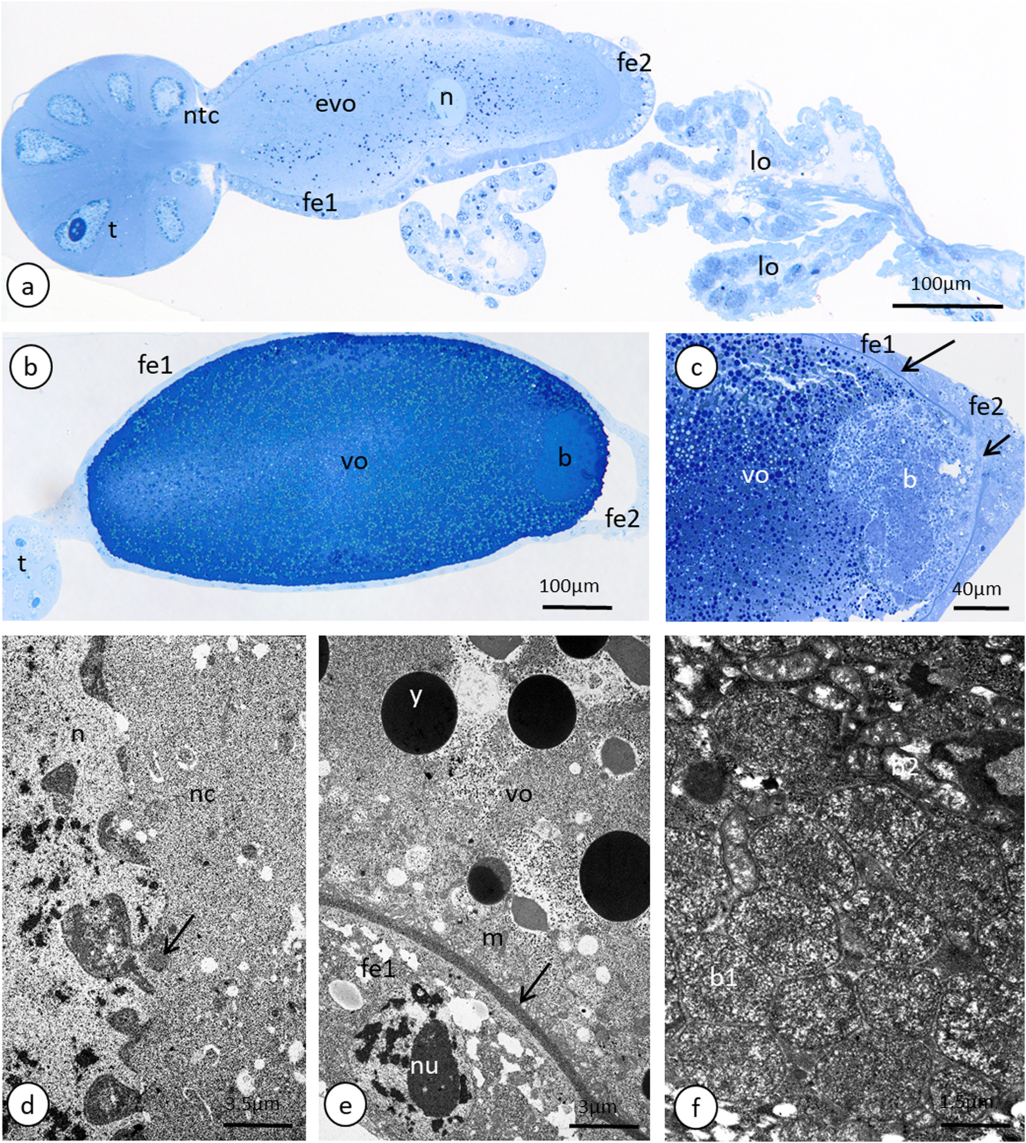
Details of the ovariole organisation and oogenesis of the oviparous female of *A. pisum*. (A) A longitudinal epoxy section stained with methylene blue through the ovariole and lateral oviducts. (B) and (C) A late vitellogenic oocyte visualised on epoxy sections. The long arrow point to the chorion, the short arrow point to the area of the oolemma that is devoid of a chorion. (D) A fragment of a nurse cell. The arrow point to accumulations of the unbounded material that is known as ‘nuage’ material. TEM. (E) A fragment of a vitellogenic oocyte and a follicular cell. The arrow point to the chorion. TEM. (F) ‘Symbiont ball’ within the vitellogenic oocyte cytoplasm. TEM. Abbreviations: b, symbiont aggregation (‘symbiont ball’); b1, bacteria of the 1st type; b2, bacteria of the 2nd type; evp, an early vitellogenic oocyte; fe1, the follicular cells that envelope the main body of the oocyte; fe2, the follicular cells at the posterior pole of the oocyte; lo, lateral oviduct; m, mitochondria; n, nucleus; nc, nurse cell; nu, nucleolus; ntc, nutritive cord; t, tropharium; vo, vitellogenic oocyte; y, yolk.

The anterior part of each ovariole is occupied by a roughly spherical tropharium, whereas the rest of the ovariole (i.e. the vitellarium) usually houses one developing oocyte that is enveloped by a follicular epithelium and a short pedicel (the basal part of the ovariole), which opens into the lateral oviduct (Figs. 5A–5D and 6A). The tropharia are composed of individual nurse cells; in the central part of each tropharium, a common cytoplasm forms the so-called trophic core, which in turn is connected to the growing oocyte via a short and broad cytoplasmic strand—the nutritive cord (Figs. 5D and 6A). At the base of the tropharia, some presumptive oocytes that do not develop (the so-called arrested oocytes) were observed (Fig. S5). Using confocal microscopy after DAPI staining, it was determined that the trophic chambers house 24 nurse cells (trophocytes). The nuclei of the nurse cells are easily detectable because they are probably highly polyploid (compare the fluorescence signals coming from nurse cells and the somatic follicular cells in Figs. 5D and 5E). The oocyte development is not synchronous, which means that the oocytes in different ovarioles are at different developmental stages, and therefore previtellogenic and vitellogenic oocytes and oocytes with fully formed eggshells can be observed in the ovarioles in the same ovary at the same time (Figs. 5A and 5B). In each ovariole, only one oocyte usually grows at a time (Figs. 5A–5D, 6A and 6B). However, in some cases between the older vitellogenic oocyte and the tropharium, a small, previtellogenic oocyte was observed (Fig. S6). Each oocyte passes through the consecutive phases of growth, gathering cytoplasm with cell organelles (previtellogenesis—Fig. S7) and then a large amount of electron-dense yolk spheres (vitellogenesis—Figs. 5A, 5B, 5D–5F, 6A–6C and 6E). During late vitellogenesis, egg envelopes composed of the vitelline envelope and chorion are produced (Figs. 6C and 6E). The posterior pole of the late vitellogenic oocytes is infested with bacteria, which form a sphere-like structure (Fig. 6C). An ultrastructural analysis revealed that two morphologically different symbionts are transmitted transovarially (Fig. 6F). The follicular cells form a simple, mono-layered epithelium. Initially, during early oogenesis (previtellogenesis, early vitellogenesis), all of the follicular cells surrounding the growing oocyte are uniform (Figs. 5D, 5E and 6A); later, they diversify into two subcategories—the cells that envelop the main body of the oocyte and the cells that cover the posterior pole of the oocyte (Fig. 6C). While both subpopulations are morphologically similar, the posteriorly located follicular cells are slightly higher than those that envelop the main oocyte body (Figs. 6A–6C). The next difference is the timing of the deposition of the chorion; the posterior cells do this later than the follicular cells that envelop the main oocyte body, which allows bacteria to invade the posterior oocyte pole (Fig. 6C).

### Oviducts, female accessory glands and spermatheca

**Figure 7:**
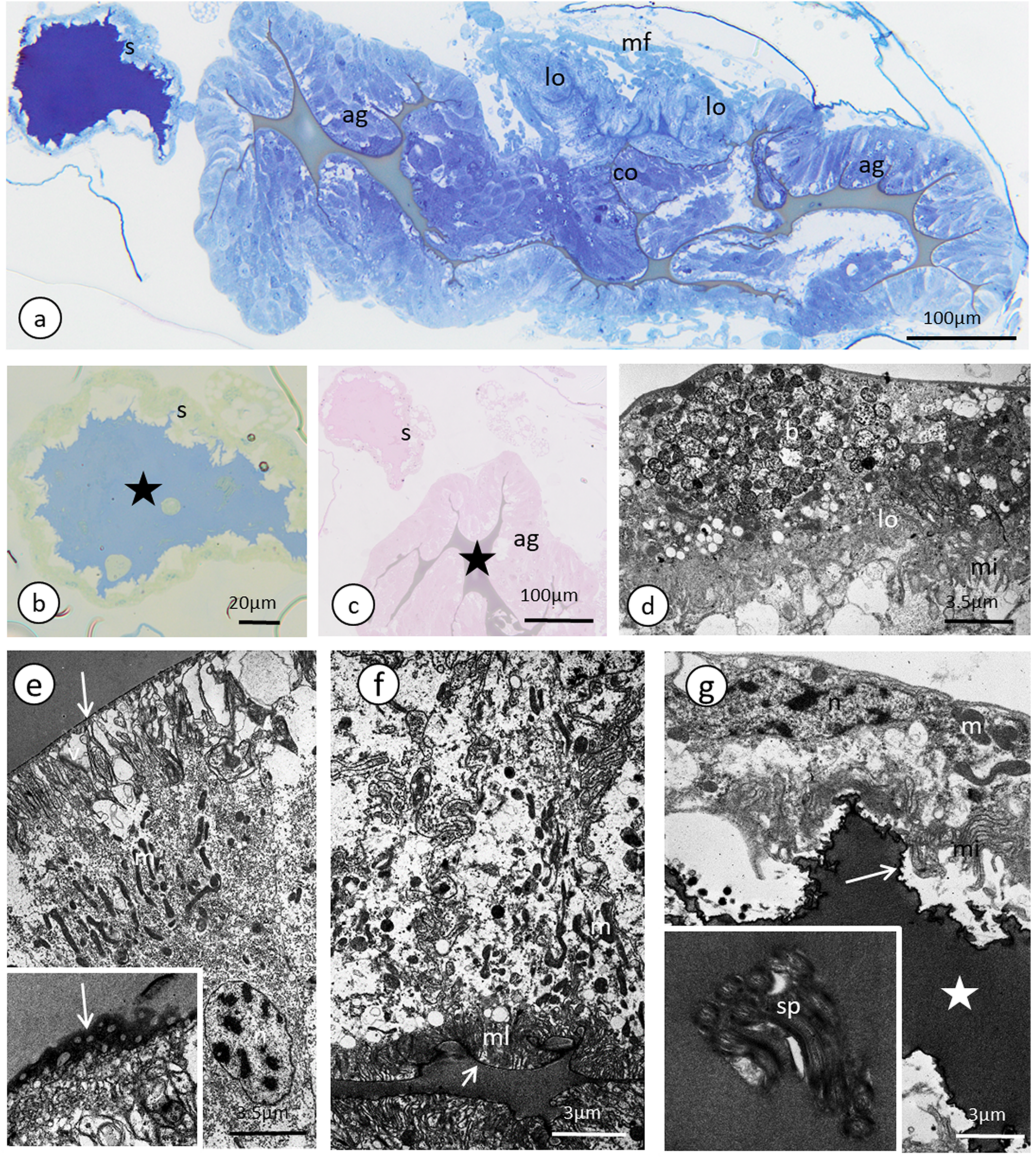
The reproductive tract of the oviparous female of *A. pisum*. (A) A semi-thin section stained with methylene blue through the lower portion of the ducts. (B) Bromophenol blue staining of the spermatheca, star—lumen with secretion. Epoxy resin semi-thin section. (C) PAS staining of a spermathecal and accessory gland, star—accessory gland lumen. Epoxy resin semi-thin section. (D) A portion of lateral oviduct. (E) and (F) Apical (E) and basal (F) portions of the epithelial cells of the accessory glands. Arrows—cuticle, the short arrows point to the basal lamina. The inset in (E) presents a higher magnification of the cuticle layer—note the interruptions. TEM. (G) Spermatheca. Arrow—cuticle, star—electron-dense substance within the lumen. Inset—spermatozoa within the spermatheca lumen. Abbreviations: ag, accessory gland; b, bacteria; co, common oviduct; lo, lateral oviduct; m, mitochondria; mf, muscle fibres; mi, microvilli; ml, membrane labyrinth; n, nucleus; s, spermatheca; sp, spermatozoa; v, vesicles.

Lateral oviducts are composed of one layer of cuboidal epithelial cells that lie on the thin basal lamina and are supported by thin muscle strands (Figs. 6A and 7D). In some lateral oviduct cells numerous bacteria occur (Fig. 7D). The apical cell membrane of cells constituting lateral oviducts forms microvilli (Fig. 7D). Some muscle fibres are loosely associated with lateral oviducts and do not form a continuous layer (Fig. 6A). The common oviduct is composed of a layer of columnar cells supported also by basal lamina and muscle fibres (Fig. 7A). The wall of each accessory gland is composed of columnar cells that lie on the thin basal lamina (Figs. 7E and 7F). The glandular cells in analysed females do not show morphological signs of high synthetic activity such as the presence of rough endoplasmic reticulum (RER) or vesicles with secretion (Figs. 7E and 7F). Instead, these cells have thread-like mitochondria scattered through the whole cell and abundant vesicles just beneath the apical plasma membrane (Figs. 7E and 7F). The apical cell surface is covered by a thin layer of cuticle (Fig. 7E). Interestingly, the cuticle is not continuous; more or less oval interruptions (‘gaps’) were observed, probably in places where vesicles export their content into the gland lumen (Fig. 7E). The lumen of glands is filled with a homogeneous substance of moderate density (Fig. 7A). Histochemical analysis showed that this substance is enriched in proteins (Fig. S8) and is also PAS positive (Fig. 7C). The wall of the spermatheca is composed of a monolayered flat epithelium (Figs. 7A and 7G). Epithelial cells lie on the thin basal lamina and their apical membranes from microvilli-like projections and are covered with a thin cuticle (Fig. 7G). The spermatheca is filled with a dense substance in which spermatozoa are embedded (Figs. 7A and 7G). As BPB staining showed, this substance is rich in proteins (Fig. 7B).

## Discussion

The Macrosiphini are the predominant group of aphids on herbaceous plants, comprising about 60% of the currently described aphid taxa (Blackman, 2010; Blackman & Eastop, 2018). They include many important pest species, but the basic knowledge about the structure of the reproductive system of sexual generation is known in only a small number of Macrosiphini (Polaszek, 1987a; Wieczorek, 2008b; Michalik et al., 2013). The pea aphid is not an exception here. Below we compare the results obtained with morphological and ultrastructural data presented to date for the reproductive systems of the sexual generation of aphids.

### Male reproductive system

Considering the number of testis follicles, the pea aphid, similarly to the majority of representatives of Macrosiphini studied so far (Polaszek, 1987a; Wieczorek, 2006), is characterised by fused testes, holding three follicles each. The vasa deferentia run independently, the accessory glands are asymmetrical and the ejaculatory duct is short (Fig. 2B). The ultrastructural organisation is simple. In particular, a layer of cuboidal or flattened epithelial cells standing on the thin basal lamina and encompassed by generally poorly developed muscles forms the walls of testicular follicles, vasa deferentia, accessory glands and ejaculatory duct (Figs. 4D–4I), as in other studied species of aphids (Wieczorek & Świątek, 2008, 2009; Vitale et al., 2009; Vitale, Brundo & Viscuso, 2011). As the male reproductive system of aphids is marked by the lack of a vesicula seminalis, the epithelial cells of the walls of the vasa deferentia and the accessory glands show secretory activity; they are rich in RER like for example, cells of vasa deferentia in *Euceraphis betulae* Koch (Vitale, Brundo & Viscuso, 2011) and epithelia of vasa deferentia and accessory glands in *Phyllaphis fagi* (L.) (Wieczorek & Świątek, 2008). However, in *A. pisum* the secretory vacuoles were not observed; probably the epithelia of male reproductive tracts in the specimens selected for ultrastructural analysis were after the period of their maximal synthetic activity. This is supported by two observations: the lumina of vasa deferentia and accessory glands were already filled with secretion and numerous epithelial cells showed some signs of degeneration such as the presence of structures similar to autophagosomes (Fig. 4H). Epithelia of testicular follicles and to a lesser extent of vasa deferentia and accessory glands form microvilli which enlarge the exchange surface between cells and the duct lumen but also suggest the phagocytic activity of these cells. In the species studied, we also observed single spermatozoa wrapped by thin protrusions of epithelial cells (for more information about phagocytic activity of male ducts, see Vitale, Brundo & Viscuso, 2011).

It is worth noting here the presence of symbiotic bacteria within *A. pisum* vasa deferentia epithelial cells. Although we found symbionts within epithelial cells only and did not notice their presence within the lumen of reproductive ducts, our ultrastructural observations are in line with the experimental results obtained by Moran & Dunbar (2006). These authors experimentally demonstrated the paternal inheritance of symbiotic bacteria in *A. pisum* and using the FISH method revealed that *Regiella insecticola* (one of the three symbionts specific for the pea aphid) was located in the testes, and especially in the accessory glands. The mechanism of symbiont transfer from males to females is not known; most likely bacteria flow together with seminal material. Hybridization experiments suggest that symbionts are absent in spermatozoa (Moran & Dunbar, 2006).

The still unresolved question is the composition of the secretion produced by the vasa deferentia and especially the accessory glands in aphids. There are morphological differences, including in *A. pisum*, in the appearance of the secretion between these two parts of reproductive ducts (see Figs. 4C, 4E and 4G; Wieczorek & Świątek, 2009). It is not known whether these differences are related to different composition, and, as a consequence, function of the secretion or the differences are only in the secretion density. Histochemical analysis showed that the secretion contains proteins and polysaccharides (Wieczorek & Świątek, 2008, 2009; present study). In other insects the male accessory glands produce components which promote sperm maturation and its nutrition and deliver other factors (e.g. regulatory peptides), which strongly influence post-copulatory behaviour, physiology of the female and fecundity (Happ, 1984; Baldini et al., 2012; Meuti & Short, 2019). Indirect evidence that this secretion can also affect the behaviour of oviparous female aphids is the rarely observed and poorly studied phenomenon of mate guarding or marking of oviparous females by males, which occurs in the unrelated species from the subfamilies Eriosomatinae and Lachninae. It is interesting that only in representatives of these subfamilies—respectively in *Pemphigus spyrothecae* Passerini (Dixon, 1998) and the genera *Cinara* and *Lachnus* (Dagg & Scheurer, 1998)—is a complete lack of accessory glands in males observed (Polaszek, 1987a). In the majority of species of aphids including *A. pisum*, males equipped with accessory glands favour a completely different type of behaviour, that is, many, rather short copulations, without any mate guarding. Moreover, male age affects the number of accessory gland cells and the quantity of produced proteins (Santhosh & Krishna, 2013). As we observed that depending on the age of the male, there is a correlation between the size of the testis follicles (smaller in older males) and the accessory glands (larger in older males) the level of secretion probably also has changed in males.

In contrast to the epithelia of the testicular follicles, vasa deferentia and accessory glands, the epithelium of the ejaculatory duct of *A. pisum* is covered by cuticle (Fig. 4I), which confirms that this part of the reproductive tract is of ectodermal origin, as is typical for insects (Chapman, 2004). The ejaculatory duct is also enveloped by a relatively well-developed layer of muscles, which undoubtedly are responsible for the force which ejects sperm.

Our observations showed that within the testes of the majority of specimens selected for ultrastructural analysis there was no spermatogenesis. In these cases within the testicular follicles we observed only some chaotically oriented spermatozoa (Fig. 5C). However, in some specimens just after the final moult, cysts with spermatids were found. These observations are in line with the earlier reports that in aphid males sperm formation occurs during larval stages (Polaszek, 1987a) or even in the embryo (Blackman, 1978). In *Glyphina betulae* (L.) and *Anoecia (Anoecia) corni* (Fabricius) only fully developed spermatozoa were found within the testes (Wieczorek & Świątek, 2009), whereas in *Phyllaphis fagi* and in five species of aphids studied by Vitale, Brundo & Viscuso (2011), testes contained developing spermatids, that is, germ cells at the final stages of spermatogenesis. To analyse the full process of sperm formation in the pea aphid we have already started ultrastructural studies of larval stages.

### Oviparous female reproductive system—ovarioles and oogenesis

We found that oviparous females have paired ovaries, each of which is composed of seven ovarioles. In each ovariole, only one oocyte usually develops at a time; however, there is no synchrony in oocyte development between neighbouring ovarioles (Figs. 5A and 5B). Similar results were presented by Bickel et al. (2013) (seven ovarioles in oviparous females), whereas Miura et al. (2003) reported six to seven ovarioles per ovary in viviparous morphs. Each ovariole is composed of an inconspicuous terminal filament, a spherical tropharium, a short vitellarium usually with one developing oocyte and a pedicel region connecting the ovariole to the lateral oviduct. This scheme of ovary organisation is typical for oviparous female aphids. However, Michalik et al. (2013) reported that in oviparous females, the number of ovarioles per ovary can vary from one to six, while from one to three oocytes can grow in a single ovary at a time. Within the tropharia, we found 24 nurse cells that seem to be polyploid. Blackman (1978) suggested that in viviparous females of the pea aphid, 32 germ cells reside, and that if the same number of germ cells is present in oviparous females, it means that eight germ cells have a potential to become an oocyte. A similar number of nurse cells (21 and 22) in the tropharia of *A. pisum* viviparous females was reported by Miura et al. (2003), which suggests that, in fact, the number of germ cells in the tropharia and the ratio of nurse cells, the presumptive oocytes, is similar in both morphs. As was found in other oviparous females of aphids (Büning, 1985; Pyka-Fościak & Szklarzewicz, 2008; Michalik et al., 2013), *A. pisum* oocytes undergo full oogenesis. Firstly, they gather cell organelles and macromolecules (in previtellogenesis), then nutrients in the form of a proteinaceous yolk (in vitellogenesis), and finally, during choriogenesis, they are covered by egg envelopes that are composed of a vitelline envelope and chorion. The same scheme of oogenesis is known from other insects that reproduce sexually (Büning, 1994).

At the posterior pole of the vitellogenic oocytes, a spherical aggregation (‘symbiont ball’) that was usually composed of two morphologically distinct symbiotic bacteria was observed (Figs. 6B, 6C and 6F). It is well known that aphid symbionts can be transmitted transovarially from one generation to another (Buchner, 1965; Baumann, 2005). In aphids, symbiotic bacteria migrate through the spaces between the follicular cells that cover the posterior pole of the vitellogenic oocyte and, after this, they immediately enter the ooplasm and form a characteristic ‘symbiont ball’ (Szklarzewicz & Michalik, 2017). During the study we did not observe the process of oocyte infestation by bacteria. However, the slightly changed morphology of the follicular cells that cover the posterior pole of the oocyte compared with those that cover the main oocyte body, the lack of the chorion on the posterior oocyte pole at late vitellogenesis, which enables symbiont transfer, as well as the presence of bacteria within the ooplasm, strongly suggest that in the pea aphid this process occurs in the same manner as in other aphids.

### Oviparous female reproductive system—genital tracts

Generally, the female genital tracts that are found in the pea aphid have the organisation that is typical for most insects; each ovariole is connected to the lateral oviduct via the pedicel region; both lateral oviducts fuse into the common oviduct that opens into the external environment through the gonopore (Chapman, 2004). The paired accessory glands and the unpaired spermatheca open into the common oviduct (Figs. 5A and 5B). All of these structures (lateral and common oviduct, accessory glands and spermatheca) are composed of epidermal cells standing on the thin basal lamina and are generally associated with a poorly developed musculature. The epithelial cells that form the walls of these ducts produce a secretion that fills their lumen and helps in transporting the egg via ducts (secretion in oviducts), helps to store the sperm (spermatheca) and enables egg adhesion to the substrate (accessory glands) (Figs. 7A–7G; Blackman, 1987; Vitale & Viscuso, 2015). Specifically, the accessory glands secrete a protective coating and adhesive substances onto the surface of the egg just before oviposition (Schmidtberg & Vilcinskas, 2016). The chemical composition of the secretion that fills the female tracts in aphids is not known; our histochemical analysis revealed that the dense secretion that fills the spermatheca and accessory glands contains proteins and is PAS positive (Fig. 7C). This secretion is produced by epithelial cells; however, in the studied specimens of the pea aphid, the secretory properties of these cells were weak (the RER was poorly developed, no Golgi complexes were detected and no secretory vacuoles with a dense content were observed, Fig. 7G). This suggests that the secretion production starts earlier, perhaps even in the larval stages, and similar to males is negatively correlated with the age of the oviparous females. The only exception was found in the accessory glands in which the epithelial cells form a columnar epithelium that is rich in mitochondria and reticulum. Moreover, the apical parts of these cells are filled with a system of irregular vacuoles that are filled with an electron-lucent content (Fig. 7E). In contrast, in *E. betulae*, numerous vacuoles with a dense or lucent secretion were found in the epithelia of the lateral and common oviducts and the spermatheca in adult oviparous females (Vitale & Viscuso, 2015), whereas the other micromorphological characters of the genital tract epithelia of *E. betulae* and the pea aphid are similar. In both cases, the epithelia lie on thin the basal lamina and their apical parts form microvilli and cell junctions in the form of zonula adherens and septate junctions were observed (Vitale & Viscuso, 2015; this study). In both species of aphids, a thin cuticle covers the epithelial cells of the common oviduct, accessory glands and spermatheca. Interestingly, the cuticle of the accessory glands in both species shows specific specialisation—it is not continuous and has numerous interruptions (Vitale & Viscuso, 2015; this study). In the pea aphid, this interruption has the form of spherical ‘gaps’ (Fig. 7C). Similar interruptions have been found in the spermathecae of some other insects (Viscuso et al., 1996; Brundo et al., 2011; Marchini et al., 2012) and, most likely, they enable the quick release of the secretion from the cells into the organ cavity.

Finally, we would like to note the presence of symbiotic bacteria in the female ducts. The bacteria that are found in the lateral oviducts of females have the same ultrastructural properties as the symbionts that are found in the male vasa deferentia (compare Figs. 4F and 7D). Usually, the symbiotic bacteria in aphids (and in other insects) are found in specialised cells that are called bacteriocytes (although some exceptions are known, reviewed by Szklarzewicz & Michalik, 2017); thus, their presence in the reproductive tracts of both the males and oviparous females suggests that they can be transmitted from one generation to offspring. However, in the light of the aforementioned experiments of Moran & Dunbar (2006), it cannot be excluded that they are transmitted from males to females.

## Conclusions

To sum up, we conclude that, histologically, the components of the reproductive system of the sexual generation of aphids are broadly similar among species studied so far, and their ultrastructure is simple. Such a simple and conservative structure of the reproductive system results from the biology of aphids. Moreover, the epithelial cells of the walls of the vasa deferentia and accessory glands of male and oviparous female *A. pisum* have secretory functions that correlate with the age of the studied morphs. Because in the temperate regions the sexual generation plays a key role in survival of aphid populations, our future work should focus on research that has the potential to improve control of these pests. In particular, characterisation of the composition and function of proteins and other molecules in the seminal fluid may help to develop methods to sterilise males and reduce the fertile population.

## Supplemental Information

10.7717/peerj.7573/supp-1 Supplemental Information 1 Raw data. A fragment of the testicular follicle with transforming spermatids—a cross section. Transmission electron microscopy.

10.7717/peerj.7573/supp-2 Supplemental Information 2 Raw data. A fragment of the follicular follicle. Epithelial cells forming follicle wall are joined via complexes of cell junctions consisting of zonula adherens and septate junctions (poorly visible). Within the follicle lumen some spermatozoa are visible.

10.7717/peerj.7573/supp-3 Supplemental Information 3 Raw data. A vas deferens wall. The singular spermatozoa are embedded in the apical cytoplasm of epithelial cells. An oblique section. Transmission electron microscopy.

10.7717/peerj.7573/supp-4 Supplemental Information 4 Raw data. A semi-thin section through vas deferens—PAS staining. Light microscopy.

10.7717/peerj.7573/supp-5 Supplemental Information 5 Raw data. An apical portion of the ovariole with tropharium, arrested oocytes and early vitellogenic oocyte. Semi-thin section stained with methylene blue.

10.7717/peerj.7573/supp-6 Supplemental Information 6 Raw data. An ovariole fragment—previtellogenic oocyte and early vitellogenic oocyte are visible. Semi-thin section stained with methylene blue.

10.7717/peerj.7573/supp-7 Supplemental Information 7 Raw data. Two ovarioles—tropharia and previtellogenic oocyte are visible. Nomarski contrast.

10.7717/peerj.7573/supp-8 Supplemental Information 8 Raw data. A semi-thin section through the accessory gland stained with bromophenol blue. Light microscopy.

## Additional Information and Declarations

### Data Availability

The following information was supplied regarding data availability: The raw data are available in Figs. 1–7 (photographs, photomicrographs and drawings) and the original, unmodified images are available as Supplemental Files.
