# Supplementary figures and images for: The reproductive system of the male and oviparous female of a model organism—the pea aphid, Acyrthosiphon pisum (Hemiptera, Aphididae)
Source: PeerJ. 2019 Sep 2;7:e7573. doi: 10.7717/peerj.7573 (PMC6727839; doi:10.7717/peerj.7573)

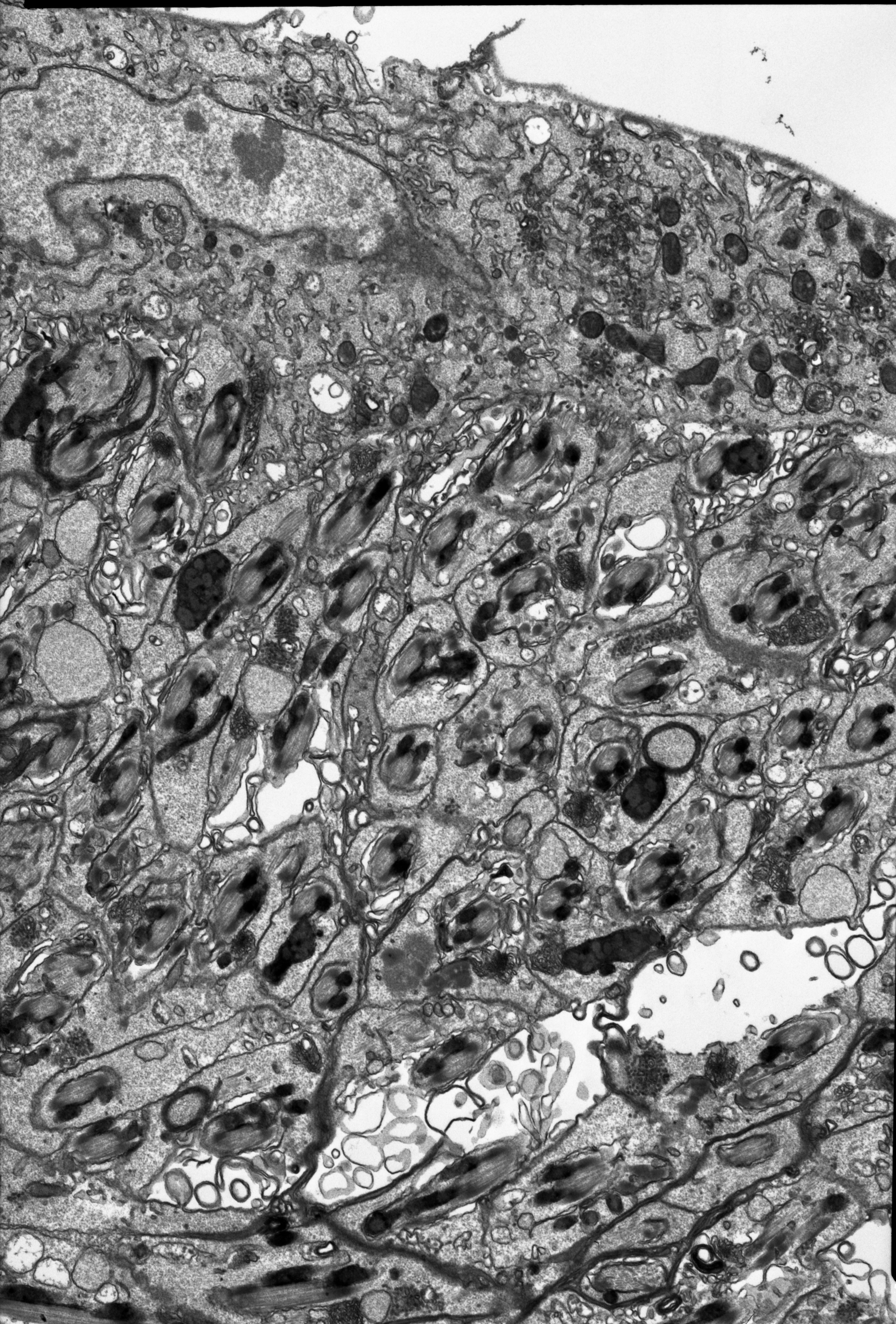

Supplement: Supplemental Information 1 [file peerj-07-7573-s001.png]

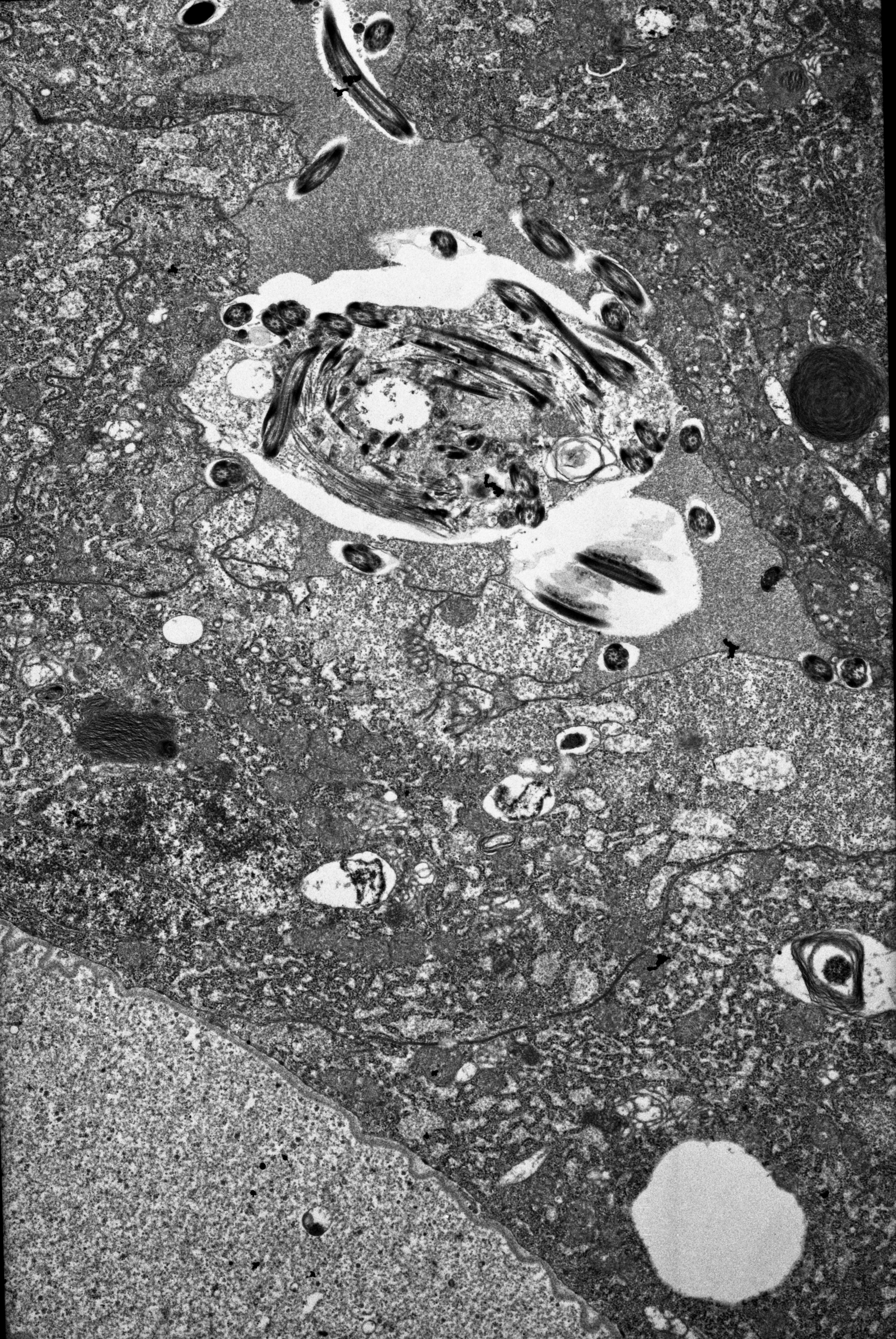

Supplement: Supplemental Information 2 [file peerj-07-7573-s002.png]

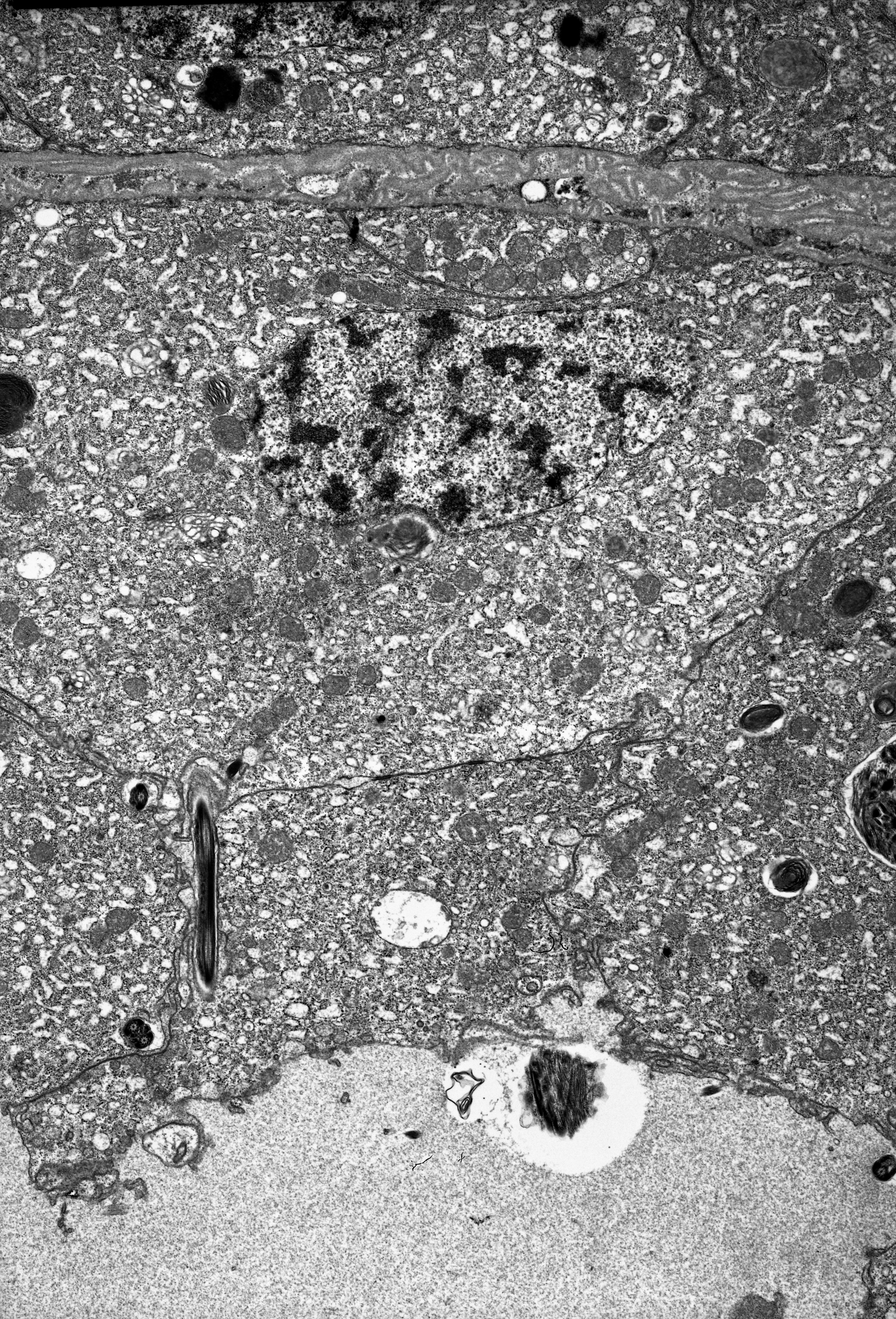

Supplement: Supplemental Information 3 [file peerj-07-7573-s003.png]

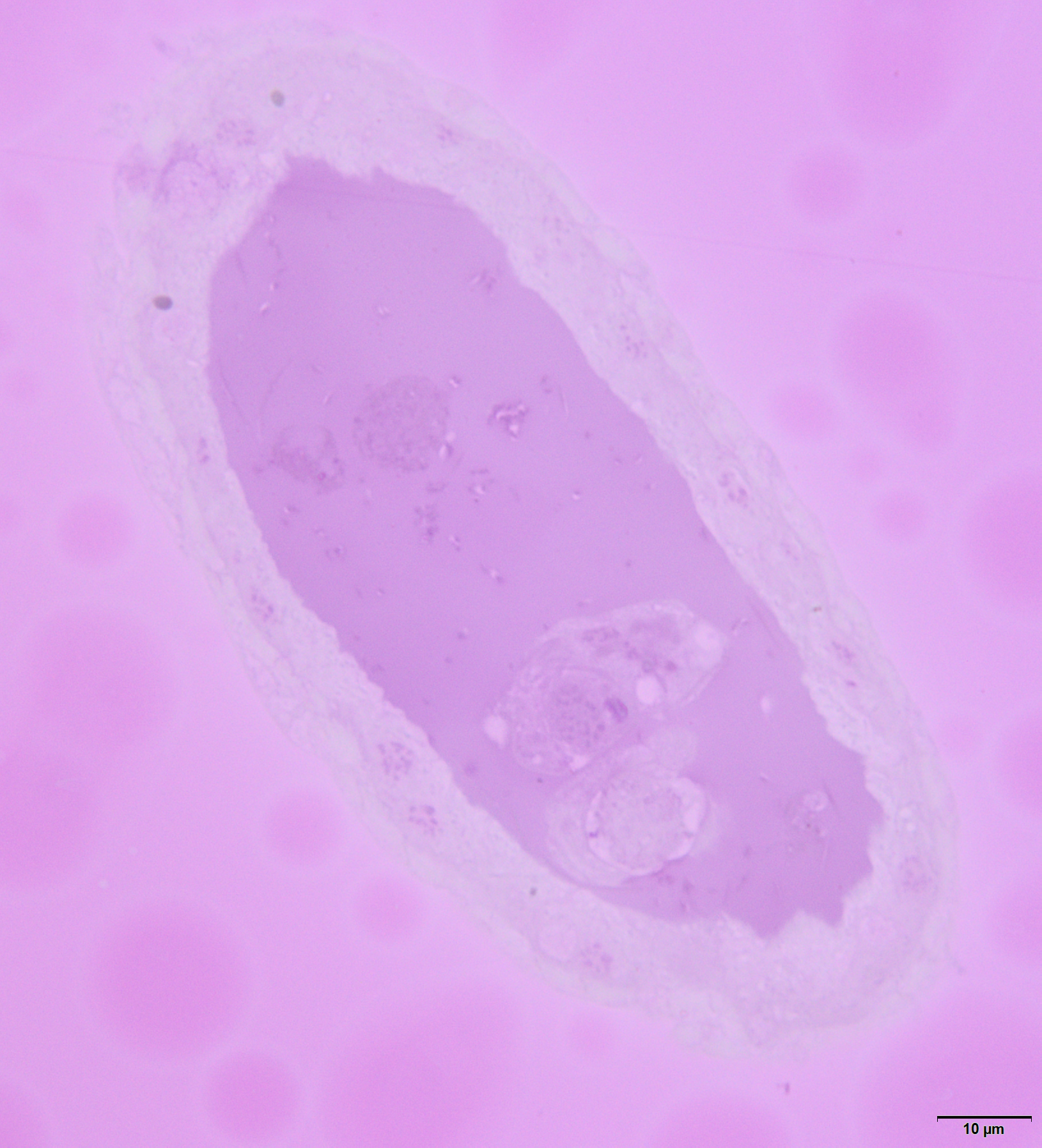

Supplement: Supplemental Information 4 [file peerj-07-7573-s004.png]

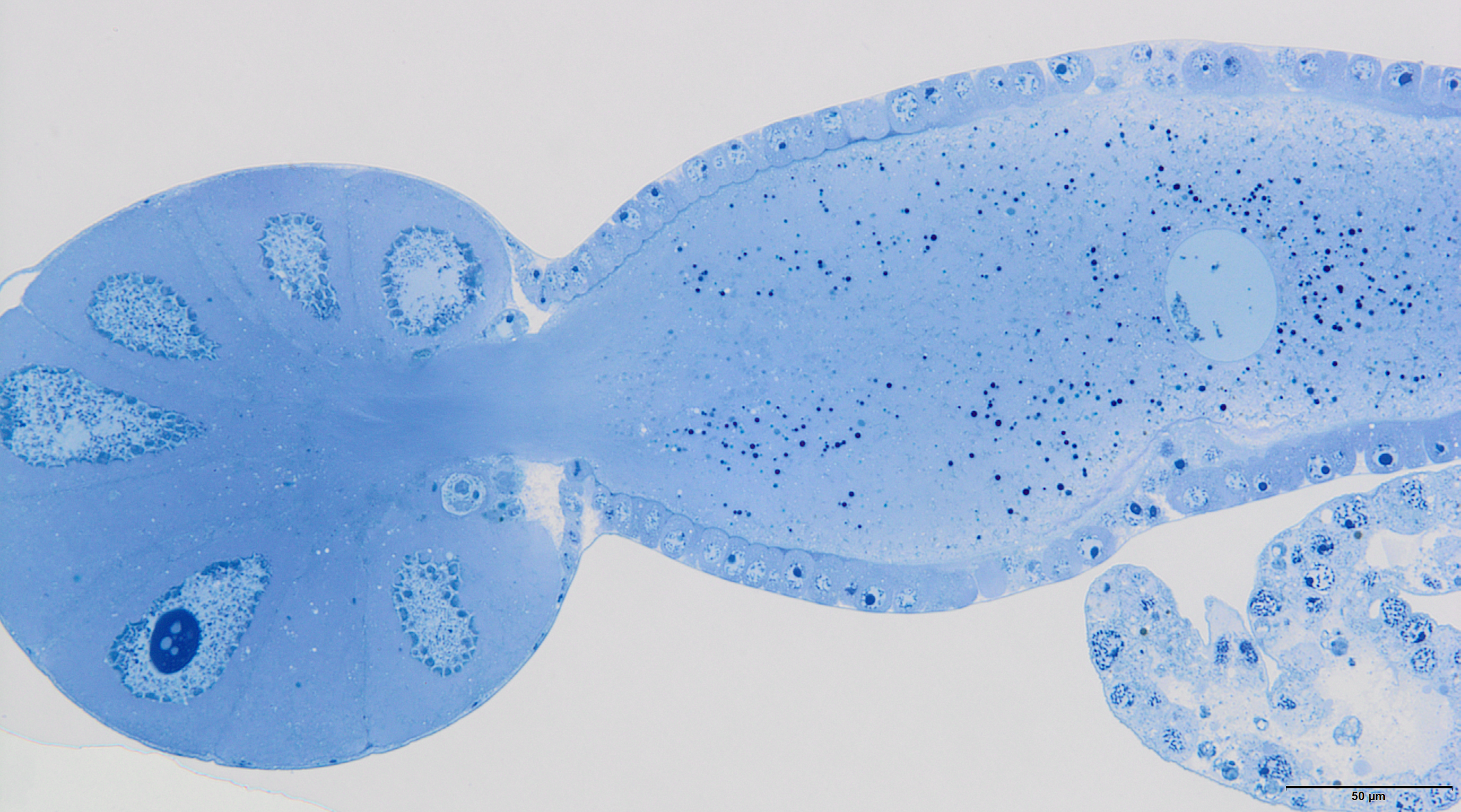

Supplement: Supplemental Information 5 [file peerj-07-7573-s005.png]

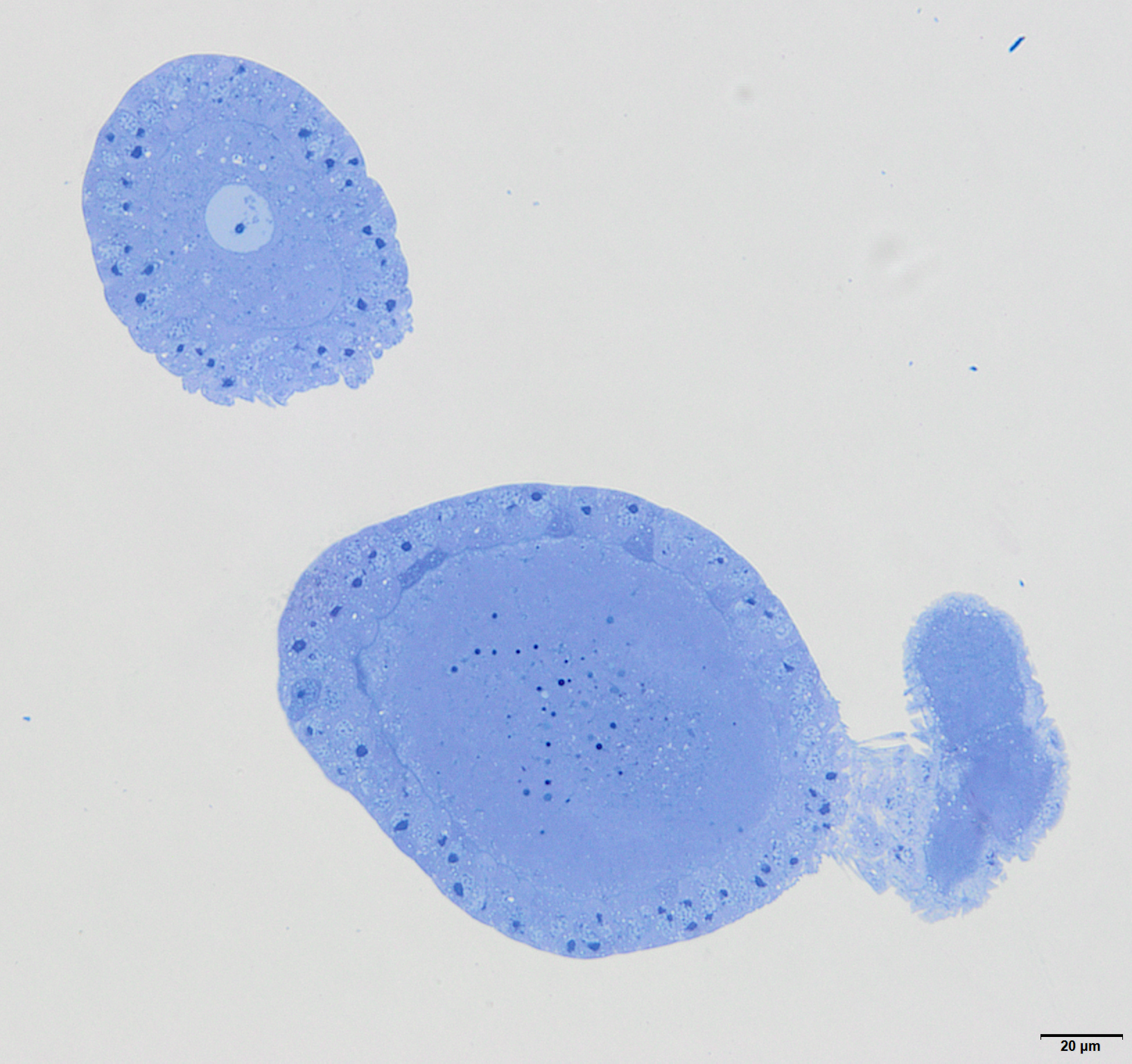

Supplement: Supplemental Information 6 [file peerj-07-7573-s006.png]

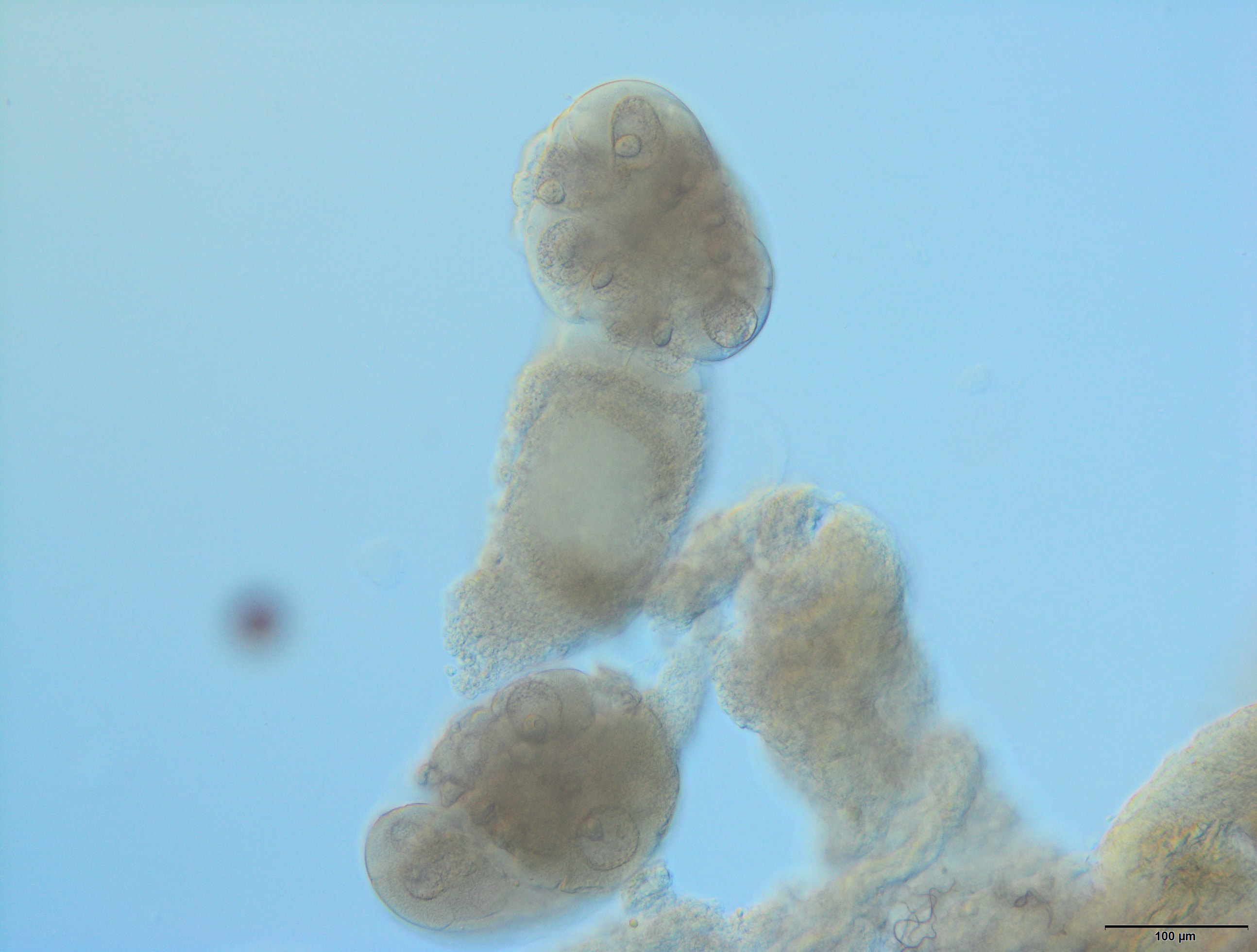

Supplement: Supplemental Information 7 [file peerj-07-7573-s007.png]

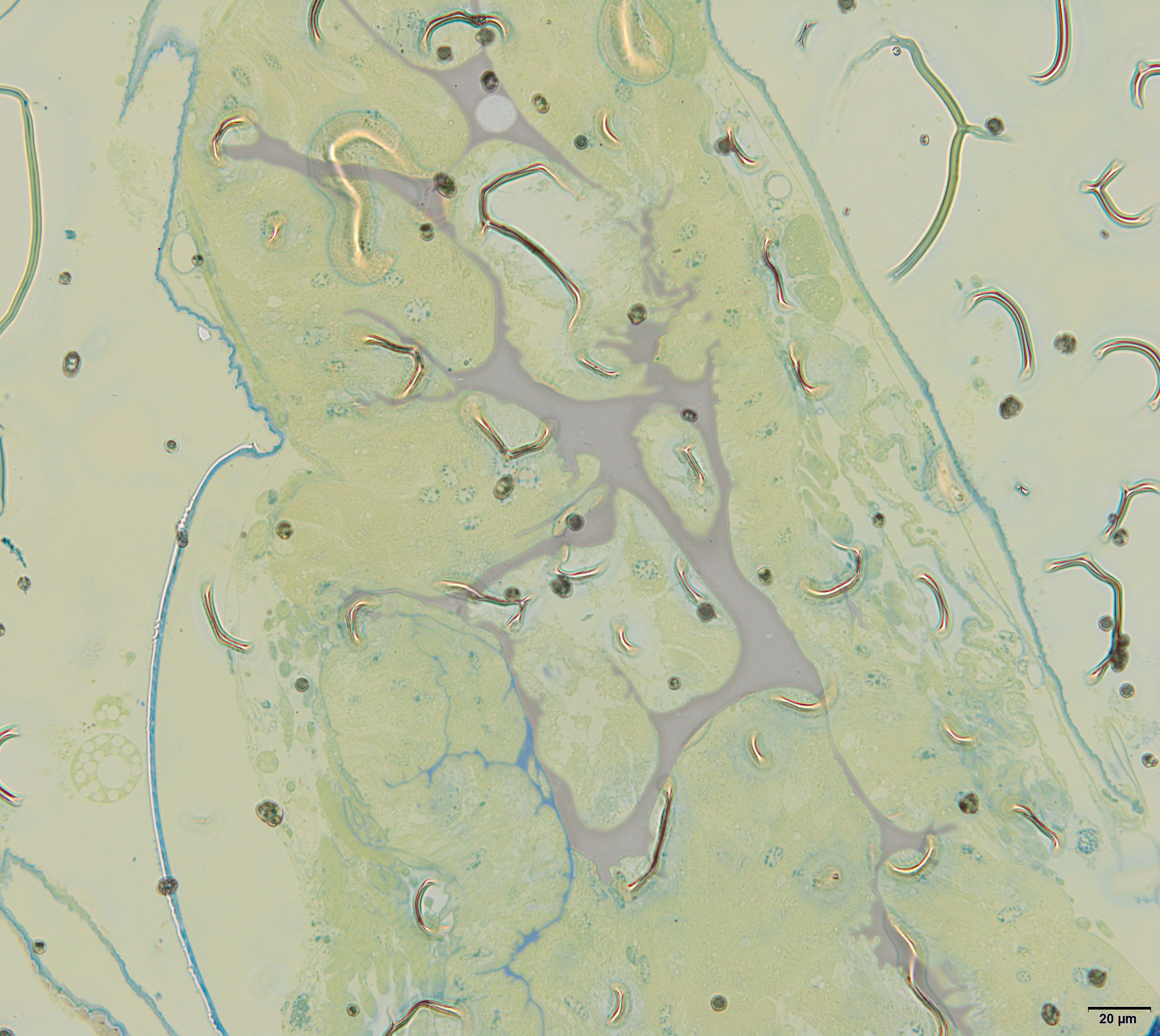

Supplement: Supplemental Information 8 [file peerj-07-7573-s008.png]
